# Determinants of undergraduate bioscience engagement: Validating attendance barriers and classroom spatial behaviour across institutions

**DOI:** 10.1002/2211-5463.70352

**Published:** 2026-10-03

**Authors:** Nigel Page, Rachel J. Hunt, Lauren J. Albee, Daniel Berwick, Alison Snape

**Affiliations:** ^1^ School of Life Sciences, Pharmacy and Chemistry Kingston University London Kingston upon Thames UK; ^2^ Faculty of Life Sciences and Medicine King's College London UK; ^3^ School of Health and Medical Sciences, City St George's University of London UK; ^4^ Present address: School of Biological and Biomedical Sciences University of Leicester UK

**Keywords:** attendance, bioscience education, classroom behaviour, learning spaces, lecture capture, student engagement

## Abstract

Student engagement with in‐person teaching remains a key challenge in undergraduate bioscience education, particularly in large‐cohort and commuter‐dominated contexts. This study evaluates a dual‐mechanism framework encompassing macro‐level structural determinants influencing attendance and micro‐level affective determinants influencing in‐class spatial behaviour. Survey data were collected from 891 undergraduate bioscience students across three London universities. Attendance barriers were assessed using ranked‐choice responses across 15 pedagogic factors and analysed using weighted priority scores. Seating behaviour was captured through self‐reported habitual classroom positioning. Non‐parametric analyses examined variation across institutional, commuting, demographic, and health‐related variables. Consistent with the hypothesis, attendance was primarily associated with macro‐level academic and structural factors, with workload management and reliance on recorded teaching emerging as the highest‐priority barriers across all institutions. Institutional differences reflected variation in lecture‐capture provision and teaching practices. Journey length was associated with academic readiness barriers, particularly for medium‐distance commuters, while demographic characteristics showed limited independent influence once structural factors were considered. In contrast, seating behaviour was strongly associated with micro‐level affective factors, particularly social anxiety and sensitivity to crowding. Students reporting these factors tended to select peripheral or low‐density areas, with minimal influence from institutional or commuting variables. These findings support a dual‐dimensional model in which attendance is shaped by structural conditions, while spatial behaviour reflects sensory and emotional factors, with implications for lecture‐capture, timetabling and inclusive learning environments in bioscience teaching.

## Graphical abstract

Student engagement extends beyond simple attendance. Across 891 bioscience students from three universities, attendance behaviour was associated mainly with structural influences, whereas classroom spatial behaviour reflected affective and sensory factors. Recognising these distinct dimensions may help institutions develop more effective timetabling, lecture‐capture policies, commuter support, and inclusive learning environments.

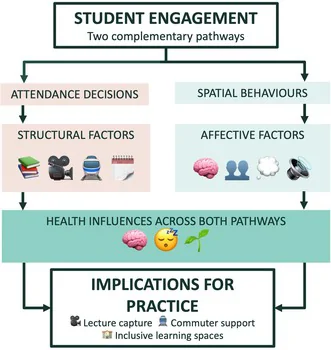

**Abbreviations**
BH: Benjamini–Hochberg (correction)
GDPR: General Data Protection Regulation
KCL: King's College London
KUL: Kingston University London
POM: Percent of Maximum
SGUL: St George's, University of London

In large‐cohort bioscience teaching environments, traditional lectures typically serve as the primary vehicle for introducing conceptually complex material that underpins subsequent laboratory and independent study. Optimising learning outcomes requires a clear understanding of how undergraduate cohorts navigate on‐campus education. This dynamic encompasses two overlapping behavioural domains: the multiple factors influencing student attendance decisions, and the spatial positioning strategies deployed by students within the physical teaching environment once present.

While in‐person attendance remains a widely used proxy for academic engagement and is positively associated with superior academic outcomes [1], tracking patterns of attendance in higher education has become increasingly complex, particularly in the post‐COVID‐19 context [2]. Rather than functioning as a direct indicator of student motivation or commitment to study, modern attendance patterns are shaped by a fluid set of strategic and contextual behaviours rather than a simple binary presence or absence. Contemporary undergraduates continuously execute cost–benefit analyses, actively balancing academic workload pressures against commuting barriers, digital learning architectures, and institutional expectations of flexibility.

A growing body of literature on awarding and participation gaps underscores the necessity of isolating systemic structural influences from individualised student variables [3, 4]. Disparities in engagement often trace back to macro‐level institutional structures, including curriculum design, assessment load, timetabling practices, and access to digital resources. The impact of these structural elements can vary across different student groups. For example, they are particularly relevant for commuting students, for whom travel time, cost, associated fatigue, and fragmented timetables can constrain available time and academic energy [5, 6, 7]. Accordingly, demographic variables, including ethnicity, were included in the present study to determine whether engagement patterns were independently associated with demographic characteristics or were better explained by underlying factors.

Within these institutional boundaries, students experience a variety of internal attendance barriers that shape their decisions around physical presence [1, 8, 9]. These barriers often manifest as responses to academic pressure, such as perceived lack of preparation, mismatches in session pace, and the deliberate prioritisation of independent catch‐up study over live lecture attendance. The increasing ubiquity of institutional lecture capture technology has fundamentally transformed this dynamic. Digital repositories permit students to weigh the pedagogical and interactive benefits of live lecture halls against the convenience of remote, self‐paced playback and the immediate mitigation of travel frictions [10, 11, 12, 13].

However, understanding whether students attend only partially captures engagement; equally important is how they position themselves within the learning space once present. Physical attendance constitutes only one dimension of undergraduate engagement. Once inside the lecture theatre, students must navigate the physical and social environment of the classroom, where seating choice impacts cognitive comfort, social proximity and visibility [14, 15]. Spatial positioning represents a behavioural mechanism through which students actively regulate their level of interaction, with some positions promoting high visibility and direct connection with academic staff, whereas others afford a strategic buffer, reduced interaction, and enhanced autonomy over the immediate sensory environment.

While some studies have considered demographic factors, there is limited consistent evidence linking seating behaviour to characteristics such as age, gender or ethnicity. Instead, spatial positioning appears to be more strongly associated with academic performance and motivational factors [14, 16, 17]. Furthermore, factors such as social anxiety and sensitivity to crowding exert significant influence on how students occupy learning spaces [18, 19].

Taken together, this landscape suggests that distinct dimensions of student engagement are governed by fundamentally separate behavioural alignments. Despite these parallel strands of research, few studies have evaluated attendance barriers and classroom spatial behaviour concurrently, or examined how institutional, structural, demographic and health‐related factors intersect across both domains. Indeed, while previous bioscience education research has separately evaluated how physical lecture theatre spatiality impacts peer dynamics and attainment [18] and how digital lecture‐capture repositories influence individual study habits [20], there remains a critical gap in understanding how these physical and digital realities actively interact. This literature gap is acutely apparent in commuter‐dominated contexts, where structural strains like journey lengths intersect with immediate, room‐level psychological pressures like social anxiety. Examining these domains side‐by‐side provides a valuable opportunity to differentiate engagement challenges that are structurally imposed from those that are intrinsically affective.

The present study addresses this gap through a multi‐institutional, hypothesis‐driven investigation of undergraduate bioscience students (*N* = 891) across three London universities featuring contrasting commuting profiles and teaching infrastructures. The study builds upon earlier work examining external structural factors shaping student engagement that included travel, timetabling, and employment [13], to explicitly contrast external engagement choices against behaviours manifested within the immediate teaching environment.

In this study, we apply a dual‐mechanism framework to test the following hypotheses: (a) macro‐level structural attendance determinants: undergraduate attendance choices are primarily dictated by systematic, structural, and logistical barriers and will operate uniformly across student demographic profiles; and (b) micro‐level affective spatial determinants: in‐class spatial positioning (seating location choice) is fundamentally associated with localised, micro‐level psychosocial and sensory‐regulation conditions (such as social anxiety and crowd sensitivity) and operates independently of macro‐level commuting constraints or institutional campus settings.

By validating these parallel behavioural pathways within a large‐scale, multi‐campus cohort, this study aims to isolate systemic variables from localised institutional cultures. In doing so, we seek to contribute a generalisable framework to guide predictive curriculum design, strategic lecture‐capture policymaking, and inclusive learning‐space architecture within modern bioscience education.

## Methods

### Research design and participants

A mixed‐methods study [21] was conducted across three London‐based universities: Kingston University London (KUL), King's College London (KCL), and St George's, University of London (SGUL; now part of City St George's, University of London). The study was quantitatively driven, with survey data providing the primary analytic strand to systematically test a dual‐mechanism model of engagement, and qualitative focus groups used to support interpretation of the findings rather than to generate parallel analyses.

An online questionnaire was administered between December 2022 and February 2023. Participants were undergraduate students across all year groups enrolled on bioscience‐related programmes and were recruited via institutionally approved communication channels, including course announcements and email distribution lists. A total of 891 students completed the survey, drawn from diverse undergraduate bioscience programmes. Participation was voluntary, anonymous, and based on informed consent.

### Data collection

The questionnaire contained 15 possible pedagogic and learning‐related attendance barriers covering workload management, preparedness, learning preferences, lecture capture, and peer and social factors. These attendance barriers were treated as independent categorical reasons for non‐attendance rather than as a psychometric scale. Students were asked to select up to three reasons that best explained occasions of non‐attendance and to rank these selections in order of importance (first, second and third). This format enabled analysis of the relative prioritisation of barriers.

Seating behaviour was assessed using a single item asking students where they ‘always or usually’ sit during on‐campus sessions, with seven response categories capturing habitual spatial positioning (e.g. front, middle, back, side/exit, low‐density areas, no typical location or wherever friends sit).

Demographic variables included gender identity, age group, ethnicity, year of study, journey length to campus, and fee status (Home/Overseas). Health‐related experiences were captured using a multi‐response checklist with an open‐ended free‐text field. Responses were grouped into seven categories: physical ill health, mental ill health, tiredness/fatigue, social anxiety/crowd sensitivity, medical appointments, disability and COVID‐related concern. All variables were coded as binary indicators (presence/absence) to evaluate their respective interactions with macro‐level barriers versus room‐level positioning.

### Statistical analysis

Attendance barriers were ranked by respondents and assigned weights (3 = first, 2 = second, 1 = third; 0 = not selected/not applicable). Where duplicate rankings occurred, only the highest‐ranked response was retained. Weighted totals were converted to Percent of Maximum (POM) scores (0–100%) to indicate relative prioritisation rather than intensity. Sensitivity analyses using alternative approaches (unweighted frequencies and inclusion of non‐unique selections) produced consistent rankings, indicating robustness.

Inferential analyses were performed on raw priority scores to statistically validate the hypotheses. Given the ordinal and non‐normally distributed data, institutional comparisons were conducted using Kruskal–Wallis tests, with Dunn–Bonferroni *post hoc* comparisons. Differences associated with binary predictors (e.g. health‐related variables) were examined using Mann–Whitney U‐tests. Multiple comparisons were controlled using the Benjamini–Hochberg procedure (Q = 0.05).

Seating behaviours were analysed using chi‐square tests of independence with standardised residuals and Cramér's V used to assess association strength. Analyses were conducted at both aggregate and institution‐specific levels.

Effect sizes were reported using epsilon squared (ε^2^), rank biserial correlation (r) and Cramér's V. ‘Prefer not to say’ responses were excluded from percentage denominators. Statistical significance was defined as *P* < 0.05 unless adjusted by correction procedures. Data preparation and visualisation were conducted in Microsoft Excel (v16.0), and statistical analyses were performed using IBM SPSS Statistics (v31.0).

### Qualitative phase and ethics

Focus groups were conducted between March and April 2023 using a semi‐structured protocol exploring learning environment experiences. Groups comprised 4–18 students and were conducted either in person or online. Participants were recruited following the survey phase through institutionally approved invitations. Participants were from the same cohort of students who had previously been invited to complete the questionnaire. Focus groups were used to support interpretation of quantitative findings and further contextualise the validated behavioural mechanisms. Participants reviewed preliminary survey results and provided reflections. Prior to participation, students were reminded of the confidential nature of the discussions and asked not to disclose information shared by other participants outside the session. Sessions were audio recorded, transcribed, anonymised, and analysed using inductive thematic analysis [22]. Following anonymisation, coding was undertaken by researchers who had not facilitated the focus groups. Themes were coded and grouped into higher‐order categories relating to structural, pedagogic, and psychosocial influences.

Ethical approval was obtained from the Kingston University's Research Ethics Committee (Reference: 3134‐SADRAS‐02) (November 2022) and the study methodologies conformed to the standards set by the Declaration of Helsinki. All participants provided informed consent, and data were handled in accordance with GDPR requirements.

## Results

### Participant characteristics

A total of 891 undergraduate bioscience students participated across three institutions: KCL (*n* = 370), KUL (*n* = 353), and SGUL (*n* = 168). The sample was predominantly female (74.2%), aged 18–21 (85.0%), and identified as Asian (46.7%), White (26.0%), Mixed/other (15.8%), and Black (11.5%). Most participants were classified as Home students (82.7%). Journey lengths to campus were categorised as Short (32.3%), Medium (45.2%), and Long (22.5%). Detailed institutional distributions are provided in Table 1.

**Table 1:** Participant characteristics by institution. Values are n (%) unless otherwise stated. Percentages for ethnicity and gender identity are calculated among respondents who selected a disclosed category. “Prefer not to say” responses are reported as counts but excluded from percentage calculations. Health‐related experiences were collected using a multi‐response item; categories are not mutually exclusive, and percentages do not sum to 100%.

Variable	Category	KCL (*n* = 370)	KUL (*n* = 353)	SGUL (*n* = 168)
Ethnicity	Asian	164 (45.9)	140 (43.2)	88 (55.3)
	Black	19 (5.3)	58 (17.9)	20 (12.6)
	Mixed/other	59 (16.5)	51 (15.7)	23 (14.5)
	White	115 (32.2)	75 (23.1)	28 (17.6)
	Prefer not to say	13	29	9
Gender identity	Female	279 (75.4)	244 (70.7)	130 (79.3)
	Male	85 (23.0)	98 (28.4)	33 (20.1)
	Non‐binary	6 (1.6)	3 (0.9)	1 (0.6)
	Prefer not to say	0	8	4
Age (years)	Under 18	3 (0.8)	1 (0.3)	0 (0.0)
	18–21	328 (88.6)	276 (78.2)	153 (91.1)
	22–25	29 (7.8)	56 (15.9)	12 (7.1)
	26–35	4 (1.1)	17 (4.8)	1 (0.6)
	36+	6 (1.6)	3 (0.8)	2 (1.2)
Year of Study	Foundation	0 (0)	61 (17.5)	0 (0)
	First	122 (33.4)	141 (39.8)	86 (51.2)
	Second	153 (41.9)	90 (25.4)	46 (27.4)
	Final	90 (24.7)	61 (17.2)	36 (21.4)
	Missing	5	0	0
Journey length to campus	Short (0–30 min)	170 (45.9)	88 (24.9)	30 (17.9)
	Medium (31–90 min)	190 (51.4)	122 (34.6)	91 (54.2)
	Long (> 90 min)	10 (2.7)	143 (40.5)	47 (28.0)
Fee status	Home	256 (69.2)	319 (90.4)	162 (96.4)
	Overseas	114 (30.8)	34 (9.6)	6 (3.6)
Health‐related experiences	Physical ill health	111 (30.0)	120 (34.0)	56 (33.3)
	Mental ill health	115 (31.1)	96 (27.2)	34 (20.2)
	Tiredness/fatigue	176 (47.6)	139 (39.4)	61 (36.3)
	Social anxiety and crowding sensitivity	70 (18.9)	66 (18.7)	30 (17.9)
	Medical appointments	35 (9.5)	61 (17.3)	24 (14.3)
	COVID‐19 related worry	23 (6.2)	11 (3.1)	11 (6.5)
	Disability	11 (3.0)	16 (4.5)	5 (3.0)
	Not applicable	110 (29.7)	102 (28.9)	56 (33.3)

### Institutional differences in attendance barriers

To investigate potential learning‐related and pedagogical attendance barriers, students were asked to select and rank up to three barriers for non‐attendance in order of priority (first, second, and third). Institutional differences in the prioritisation of these attendance barriers were examined using Kruskal–Wallis tests on the priority scores (0–3). Several high‐priority barriers showed statistically significant differences between institutions, with the largest effect observed for the availability of recordings (ɛ^2^ = 0.07), reflecting the absence of institutional lecture capture provision at KUL at the time.

Importantly, while statistically significant differences between institutions were detected for several attendance barriers, the relative prioritisation of key barriers was largely stable (Table 2). Institutional effects generally reflected differences in magnitude rather than qualitative shifts in which barriers were most prominent. This consistency aligns with the descriptive Percent of Maximum (POM) comparisons, which showed a similar rank ordering of primary barriers across all institutions.

**Table 2:**
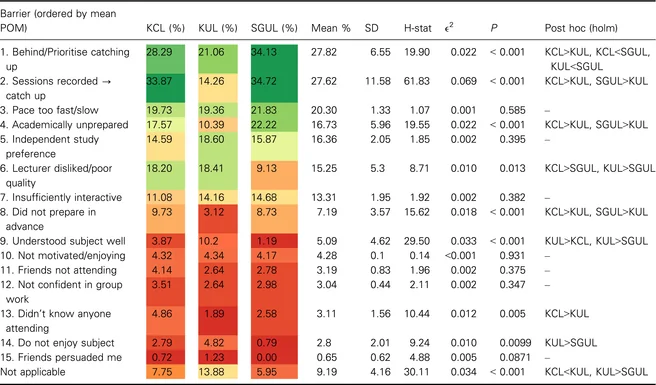
Institutional comparison of attendance barriers, ordered by mean Percent of Maximum (POM) endorsement. Institutional differences were tested on the underlying ranked response data using Kruskal–Wallis tests with Holm‐corrected Dunn *post hoc* comparisons. Heat map colour intensity reflects percentage endorsement, with green shading indicating higher levels of endorsement and red shading lower levels of endorsement; darker shades indicate greater magnitude.

Significant institutional effects were observed for being behind with studies, sessions being recorded, and academic preparedness (all *P* < 0.001), as well as lecturer‐related factors (*P* = 0.013). Post hoc Dunn tests indicated that these differences were driven by specific institution contrasts rather than consistent gradients across all three groups. For example, prioritisation of being behind with studies was significantly higher at SGUL compared with both KCL and KUL, whereas prioritisation of sessions being recorded was significantly lower at KUL than at the other institutions. This pattern in lecture‐capture appears to correspond to differences in provision across the institutions at the time of data collection, with KCL and SGUL operating institution‐wide lecture‐capture systems, whereas KUL did not have a universal lecture‐capture policy. Despite these differences, the overall ranking of high‐priority barriers was consistent across institutions, validating that academic backlog, access to recorded sessions, and session pace remained universally prominent.

The attendance barriers related to social and contextual factors did not differ significantly between institutions; however, social familiarity (not knowing anyone attending) showed a significant institutional effect (*P* = 0.005), primarily driven by higher prioritisation at KCL compared to KUL. In contrast, other moderate‐to‐low priority barriers including preference for independent study (*P* = 0.396), session interactivity (*P* = 0.382), peer attendance (*P* = 0.375), and confidence in group work (*P* = 0.347) did not differ significantly between institutions (*P* > 0.05).

### Journey length and attendance barriers

The survey asked students to select the duration of their typical journey to campus. Journey length was significantly associated with several attendance barriers, particularly those relating to academic readiness and study strategy (Table 3). As illustrated in Fig. 1, differences in prioritisation were observed by travel time. Students with journeys of 31–90 min reported higher priority scores for feeling academically unprepared (H = 12.30, *P* = 0.032, ɛ^2^ = 0.014) and not preparing in advance (H = 11.02, *P* = 0.032, ɛ^2^ = 0.012) compared to long‐journey groups. These effects were small but consistent across institutions.

**Table 3:** Attendance barriers by journey length (N = 891). Journey groups were S = Short (< 30 min), M = Medium (31–90 min), L = Long (> 90 min). Pooled Trend indicates the order of mean priority scores (weighted 3–2‐1). H = Kruskal–Wallis statistic; ɛ^2^ = effect size. *P*‐values are Benjamini–Hochberg adjusted; *denotes *P* < 0.05. Institutional Peak indicates the journey group with the highest mean score for KCL, KUL, and SGUL, respectively.

Barrier	Pooled trend	H(2)	ɛ^2^	BH‐adj p	Post hoc trends	Institutional peak (KCL | KUL | SGUL)
1. Behind/Catching up	M > S > L	5.33	0.006	0.16	ns	L | M | S
2. Sessions recorded	M > S > L	2.10	0.002	0.401	ns	L | L | L
3. Pace fast/slow	L > M > S	3.61	0.004	0.328	ns	S | L | L
4. Academically unprepared	M > S > L	12.30	0.014	0.032*	M > L	M | S | M
5. Independent study	L > M > S	8.96	0.010	0.048*	L > S, L > M	L | L | L
6. Lecturer quality/disliked	S > L > M	7.53	0.009	0.074	S > M	L | M | S
7. Insufficiently interactive	L > S > M	1.62	0.002	0.474	ns	S | L | S
8. Did not prepare in advance	M > S > L	11.02	0.012	0.032*	M > L	M | M | M
9. Understood subject well	L > S > M	2.65	0.003	0.389	ns	S | M | M
10. Not motivated/enjoying	S > M > L	3.11	0.004	0.377	ns	S | S | S
11. Friends not attending	S > M > L	7.61	0.009	0.074	S > M	S | S | L
12. Not confident in group work	M > L > S	2.45	0.003	0.392	ns	L | S | L
13. Didn't know anyone attending	M > S > L	2.87	0.003	0.381	ns	M | M | M
14. Do not enjoy subject	S > L > M	6.94	0.008	0.082	S > M, L > M	L | S | L
15. Friends persuaded me	S > L > M	0.82	0.001	0.662	ns	S | S | S
16. Not applicable	S > L > M	2.10	0.002	0.401	ns	S | S | S

**Fig. 1:**
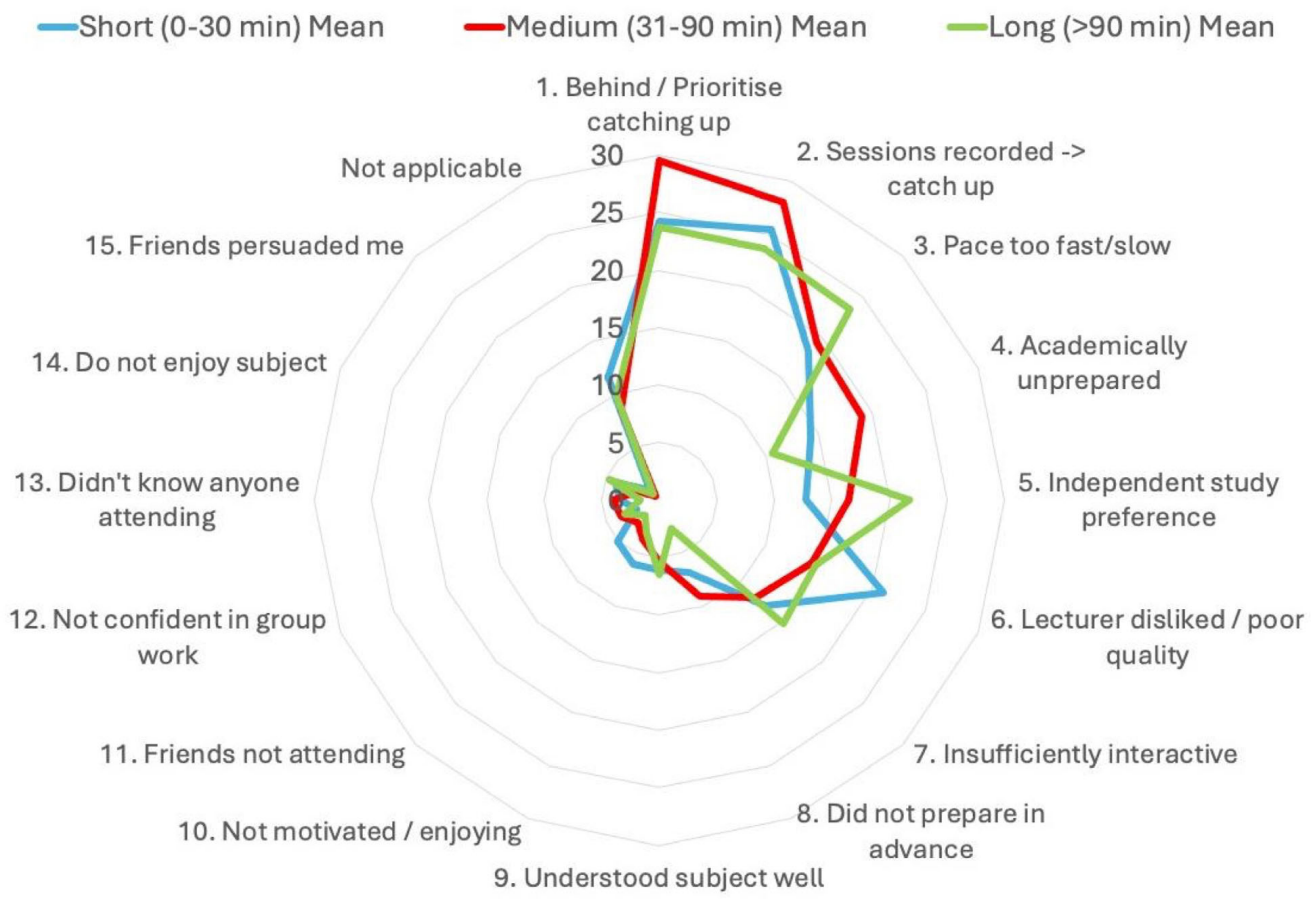
Radar chart of attendance barrier profiles by journey length, ordered by mean percentage of maximum (POM) endorsement.

Preference for independent study followed a linear pattern (Long > Medium > Short), with long‐journey students showing significantly higher prioritisation than both other groups (*P* = 0.048, ɛ^2^ = 0.010). This pattern corresponded to higher prioritisation of independent study among students with longer commutes. While short‐journey students (0–30 min) showed higher mean scores for lecturer‐related dissatisfaction and certain social barriers (e.g. friends not attending), these did not reach statistical significance after adjustment (*P* = 0.074).

No significant differences were observed for motivation, perceived session pace, or reliance on recorded teaching (*P* > 0.05), indicating broadly similar patterns across journey groups. Institution‐specific analyses showed that preparation‐related effects were consistent across sites. Reliance on recorded teaching varied in magnitude between institutions but was highest among long‐journey students in all cases.

Overall, journey length was associated with differences in the intensity of barrier prioritisation rather than qualitative differences in barrier type.

### The interplay of demographics and commute

Ethnicity was significantly associated with journey length (χ^2^(2, *N* = 840) = 50.32, *P* < 0.001), with minority ethnic students more frequently represented in medium‐ and long‐journey cohorts. White students were more likely to reside within 30 min of campus. Once stratified by journey length, ethnicity showed limited independent association with attendance‐barrier profiles. Mean priority scores indicated that the primary drivers Behind/Catching Up and Sessions Recorded remained consistent across ethnic groups within journey‐length categories.

Preference for independent study increased with travel time across all ethnic groups. Stratified analyses did not identify statistically significant ethnicity‐based differences for primary attendance barriers after adjustment. The only notable exception was Confidence in Group Work, where differences were observed within the long‐journey cohort (*P* = 0.0007). Specifically, White students with long commutes were the most likely to report group work anxiety as a barrier (POM = 12.22%), whereas its relevance dropped to near‐zero for Asian (1.06%) and Black students (0.00%). Overall, attendance‐barrier profiles were broadly consistent across ethnic groups once accounting for journey length.

### Additional demographic predictors

Attendance‐barrier profiles were largely consistent across gender, age group, and Home/Overseas status, and year of study. Academically oriented barriers and reliance on recorded teaching showed the highest levels of endorsement across all groups. Residency status (Home/Overseas) was initially associated with subject enjoyment (*P* = 0.050); however, this association appeared to be explained by differences in journey length, with overseas students reporting shorter commutes (χ^2^(2, *N* = 891) = 72.24, *P* < 0.001). Once stratified, no significant differences remained, validating the primacy of the structural commute variable.

Gender, age and year of study showed independent associations not explained by commute patterns. Female students showed greater sensitivity to session pace (*P* = 0.020), while younger students (18–21) prioritised social factors, such as friends not attending (*P* = 0.022), more than older students. Furthermore, academic progression from foundation to final year introduced a distinct increase in concerns regarding catching up (*P* = 0.008), whereas early‐stage confidence in understanding the subject declined (*P* < 0.001). Aside from these specific differences, attendance barriers were broadly consistent across demographic groups.

### Health‐related attendance barriers

Of the seven self‐reported health‐related factors analysed, each coded as a binary indicator (present/absent), two (social anxiety/crowding sensitivity and tiredness/fatigue) showed significant associations with specific attendance barriers following Benjamini–Hochberg correction (*P* < 0.05, Table 4, Fig. 2). These factors were small but consistent. Students reporting social anxiety and crowding sensitivity showed lower confidence in group work (*P* = 0.037, *r* = −0.09) and higher prioritisation of independent study (*P* = 0.043). Students reporting tiredness/fatigue showed higher endorsement of not knowing anyone attending (*P* = 0.043). Other health factors, including mental health, physical health, disability, and COVID‐related concerns, were not significantly associated with specific barriers. Overall, health‐related factors were associated with specific social and attendance‐related barriers rather than general attendance patterns.

**Table 4:** Significant associations between health‐related factors and attendance barriers (*N* = 891) assessed using Mann–Whitney U tests followed by Benjamini–Hochberg correction (*P* < 0.05). Sub‐sample sizes for health factors are social anxiety (*n* = 166) and tiredness (*n* = 376). Means (M) represent weighted barrier scores (3–2–1) for students reporting the health factor (S) versus those not reporting it (NS). Effect sizes (*r*) indicate the direction and magnitude of association; negative values correspond to higher barrier endorsement among students reporting the health factor.

Health factor & associated attendance barrier	M (S/NS)	*r*	BH‐corrected p	Interpretation
Social Anxiety and Crowding Sensitivity				
Not confident in group work	0.24/0.06	−0.09	0.037	Increased avoidance of collaborative learning environments
Prefer independent study	0.65/0.46	−0.12	0.043	Stronger preference for solitary learning
Tiredness/Fatigue				
Didn't know anyone attending	0.16/0.05	−0.05	0.043	Fatigue lowered threshold for social withdrawal

**Fig. 2:**
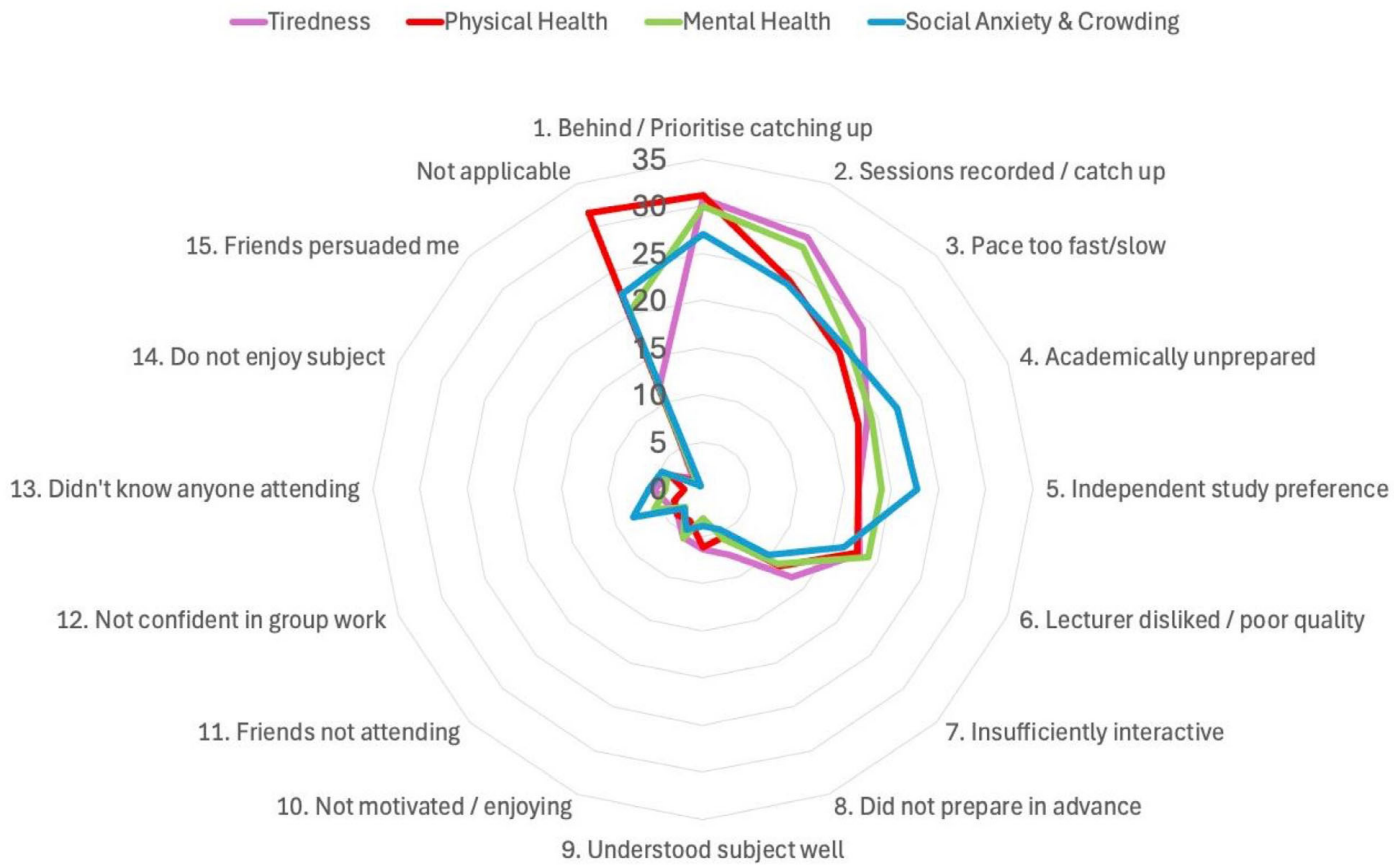
Radar chart of the four most significant health‐related factors by barrier prioritisation, ordered by mean percentage of maximum (POM) endorsement.

### Summary of attendance barrier patterns

Across analyses, attendance barriers were most frequently associated with academic workload and access to recorded teaching, with similar ranking patterns observed across institutions and demographic groups. Journey length and institutional context showed the strongest associations with variation in barrier prioritisation, particularly for academically oriented barriers. Increasing year of study introduced developmental shifts, marked by increasing catch‐up pressures and a decline in confidence. Health‐related factors were associated with selected social and attendance‐related barriers. Gender, ethnicity, and residency status showed limited independent effects once structural factors were considered. While minor differences were observed for gender (sensitivity to session pace) and age (social motivation), overall patterns of attendance barriers were consistent across demographic groups (Table 5).

**Table 5:** Summary of the relative influence of predictors on attendance barriers assessed using Kruskal–Wallis tests (for multigroup variables such as journey length and institutional context), Mann–Whitney U‐tests (for binary health variables) and Chi‐square tests (for categorical demographics) with Benjamini–Hochberg corrections, where applicable. Influence levels reflect relative explanatory strength based on effect size and consistency across analyses, rather than a formal or statistical ranking.

Relative association	Factor	Statistical status	Primary impact area	Key finding
Higher association	Journey Length	Significant (*P* < 0.001)	Academic readiness	Most consistent predictor of barrier variation; medium‐distance commuters reported the highest academic strain (e.g. Behind/Unprepared)
Moderate association	Institutional Context	Significant (*P* < 0.001)	Teaching environment	Institutional practices regarding recorded teaching shaped responses to workload‐related pressures.
Moderate association	Year of Study	Significant (*P* < 0.01)	Curriculum workload	Progression brought higher catch‐up pressures, alongside declining confidence
Moderate association	Health (anxiety/crowding, fatigue)	Significant (*P* < 0.05 after BH correction)	Social–sensory barriers	Anxiety and crowding sensitivity were linked to increased group‐work barriers; fatigue was linked to social uncertainty.
No significant association	Demographics (Ethnicity, residency, age, gender)	Non‐significant (structural) to significant (behavioural)	Structural proxy and specific variations	Ethnicity and residency primarily functioned as proxies for journey length. Once stratified, academic barrier profiles were universal. Behavioural exceptions included session pace by gender and social motivation by age (p < 0.05)

### Overview of seating behaviours across institutions

Students reported their usual seating location within on‐campus teaching sessions by selecting one of seven predefined categories across the three participating institutions (Table 6). The most frequently selected positions were the middle of the room (32.0%), front seating (16.5%), and social seating (‘wherever my friends sit’; 15.9%). A smaller proportion consistently selected back‐row seating or reported no typical location, while peripheral seating (side or exit‐adjacent) was selected by 5.6% of participants. Distributions were similar across institutions.

**Table 6:** Values are *n* (%). Percentages represent the proportion of students within each institution selecting a given typical seating location. “Where my friends sit” indicates students who prioritise proximity to peers over a specific physical area of the room.

Seating location	KCL *n* (%)	KUL *n* (%)	SGUL *n* (%)	Total *n* (%)
Always/usually at the back	48 (13.0)	34 (9.6)	29 (17.3)	111 (12.5)
Always/usually at the front	54 (14.6)	63 (17.8)	30 (17.9)	147 (16.5)
Always/usually in the middle	119 (32.2)	113 (32.0)	53 (31.5)	285 (32.0)
Always/usually near the side/exit	24 (6.5)	14 (4.0)	12 (7.1)	50 (5.6)
Section with the fewest people	18 (4.9)	17 (4.8)	3 (1.8)	38 (4.3)
No typical location	53 (14.3)	46 (13.0)	19 (11.3)	118 (13.2)
Wherever my friends sit	54 (14.6)	66 (18.7)	22 (13.1)	142 (15.9)

### Institutional comparison of seating behaviour

A chi‐square test of independence showed no statistically significant association between institution and seating behaviour (χ^2^(12) = 16.52, *P* = 0.168), with a negligible effect size (*V* = 0.096).

### Demographic predictors

Ethnicity, journey length, year of study and Home/Overseas status were not significantly associated with seating behaviour (*P* > 0.05), and effect sizes were very small (Cramér's *V* ≤ 0.10). Seating distributions were broadly similar across ethnic groups, commute length categories, and fee‐status groups, although isolated institution‐specific associations were observed. Crucially, the lack of association with commute length indicates that macro‐level logistical factors did not significantly predict in‐class spatial positioning within this dataset.

Gender and age showed statistically significant associations with seating choice. Gender differences were distinct (χ^2^(6) = 27.29, *P* < 0.001, V = 0.177): female students were significantly more likely to occupy central or front‐row seating (50.7% combined). Conversely, male students were more likely to select peripheral or low‐density locations, specifically showing a three‐fold higher tendency than females to seek out the ‘section with the fewest people’ (8.3% vs 2.8%).

Age also showed a significant association (*V* = 0.147), driven by the marked over‐representation of older students (26 and over) in front‐row seating; while only 13.9% of the youngest cohort (21 and under) sat at the front, 63.6% of students aged 26+ occupied these seats. These patterns showed some institutional variation and were not uniformly replicated across all institutions.

Institution‐specific analyses identified different patterns of association across sites. At KCL, seating behaviour was associated with gender and residency status, whereas at KUL, associations were observed with ethnicity and age. These patterns were not consistently observed across all institutions.

### Health‐related predictors of seating behaviour

Health‐related factors showed the strongest associations with seating behaviour. Social anxiety/sensitivity to crowding (χ^2^(6) = 69.07, *P* < 0.001, *V* = 0.278) showed the largest effect on seating choice, with an effect size nearly double those of demographic factors such as age or gender. This factor was associated with a dramatic shift from central to peripheral locations: students sensitive to social anxiety/crowding were five‐and‐a‐half times more likely to select a seat in a section with the ‘fewest people’ (~ 12.7% vs. ~ 2.3%) and twice as likely to sit in the back row (~ 20.5% vs. ~ 10.6%) compared to the general population. Mental ill health also showed a highly significant association with seating choice (χ^2^(6) = 27.00, *P* < 0.001, *V* = 0.174), specifically driving a preference for ‘Side or Exit’ seating (~ 9.4% vs ~ 4.2%) and the ‘Section with the fewest people’ (~ 8.2% vs ~ 2.8%).

In contrast, physical ill health, tiredness/fatigue, medical appointments, and COVID‐related worry showed no significant associations with seating location (*P* > 0.05, *V* < 0.11). Associations were stronger for psychological and sensory factors than for physical or logistical health factors. Finally, disability occupied a borderline statistical position (χ^2^(6) = 12.54, *P* = 0.051), suggesting a non‐significant trend toward peripheral seating while otherwise mirroring the ‘middle‐row’ dominance of the holistic sample.

### Summary of seating behaviour patterns

Across aggregate and institution‐level analyses, seating behaviour showed no significant association with institutional context, ethnicity, journey length, fee status or year of study (*V* < 0.10, *P* > 0.05). Gender and age were associated with statistically significant differences, although effect sizes were small to moderate (*V* < 0.15) and not consistently observed across all institutions. In contrast, health‐related factors, particularly social anxiety and sensitivity to crowding, showed the strongest associations with seating behaviour (*V* ≈ 0.28). Students reporting these factors were more likely to select peripheral or low‐density seating locations and less likely to occupy central areas (Table 7).

**Table 7:** Relative strength and consistency of associations between student characteristics and seating behaviour based on Chi‐square tests and Cramér's V. Influence levels reflect relative explanatory strength based on effect size and consistency across analyses, rather than a formal or hierarchical statistical ranking.

Relative association	Factor	Statistical status	Effect size	Key interpretation
Higher association	Health (Social Anxiety)	Strong & consistent (*P* < 0.001)	Moderate (*V* ≈ 0.28–0.29)	Marked preference for peripheral, low‐density seating; strong avoidance of the central ‘middle’ zone.
Moderate association	Age	Highly Significant (*P* < 0.001)	Small‐Medium (*V* ≈ 0.20)	Marked transition to front‐row seating as age increases.
Moderate association	Mental Ill Health, Gender	Significant (*P* ≤ 0.001)	Small‐Medium (*V* ≈ 0.17)	Drives central vs. peripheral splits; males significantly more likely to seek ‘low‐density’ areas.
No significant association	Institution, Ethnicity	Non‐significant (*P* > 0.05)	Negligible (*V* < 0.10)	Seating distributions remain broadly stable across campuses and ethnic groups.
No significant association	Home/Overseas, Journey Length/Year of Study	Non‐significant (*P* > 0.05)	Negligible (*V* < 0.08)	Neither domicile nor commute duration had any predictive power over a student choice of seating.

### Qualitative analysis of student attendance and engagement

Qualitative analysis identified four themes that describe how students navigate logistical constraints, pedagogical value, and academic workload when deciding whether to attend on‐campus teaching.

#### Commuting demands and timetabling friction

Students frequently described attendance decisions as contingent on the perceived proportionality between travel effort and session duration. Medium‐length commutes were often characterised as too long to feel convenient, yet not long enough to justify full disengagement from campus. One student described this calculus as follows: ‘If I'm coming in just for an hour, is it worth the 45‐minute train ride and the money?’ [P7].

Fragmented timetables further reduced willingness to attend, particularly when sessions were separated by long gaps. As one participant noted, ‘You've got three‐hour lectures and then five hours gap. You're just wasting that time’ [P3].

#### Use of recorded teaching for academic pacing

Students described recorded teaching as a routine component of their study practices, particularly where live delivery was perceived as too fast. Participants highlighted the ability to pause, replay, and selectively review content as a key advantage: ‘I can replay that bit over and over again until I actually understand the concept’ [P12].

Several students also reported adapting their engagement strategies over time, particularly in later years of study, using recordings to manage workload more efficiently. One participant explained: ‘I watch it at 1.5 or two times speed because now I'm better at identifying what's important’ [P1].

#### Perceived value of different teaching formats

Participants distinguished between teaching sessions they perceived as more or less valuable to attend in person. Interactive sessions were reported as easier to sustain attention in, with quizzes or breaks between content segments helping to maintain engagement: ‘It makes you stay awake’ [P9].

Attendance was also influenced by proximity to assessment. One student noted increased attendance at lectures directly linked to upcoming coursework: ‘That's why everyone goes to that one’ [P4]. In contrast, seminars and tutorials were frequently described as offering opportunities for collaborative discussion: ‘You absorb knowledge from other people as well’ [P10].

#### Managing the experience of ‘being behind’

A recurring feature of the data was the experience of cumulative academic pressure associated with clustered deadlines. Students described periods of low assessment demand followed by simultaneous submissions, leading them to prioritise completing existing work over attending new sessions. One participant commented: ‘Nothing is assigned for months and then everything comes at once’ [P14].

Some students expressed a preference for regular, low‐stakes assessment structures that would help regulate engagement with content across the semester. As one participant suggested, ‘Constant quizzes every week… involving active recall’ [P13].

## Discussion

This study examined two complementary dimensions of student engagement: attendance barriers influencing attendance decisions and classroom seating behaviour reflecting in‐class spatial behaviour. By analysing these domains in parallel, the findings indicate two analytically distinct patterns of engagement behaviour. Student attendance decisions were primarily associated with academic workload pressures and structural conditions, whereas seating behaviour was more strongly associated with sensory and emotional factors. Across both domains, demographic characteristics showed limited independent influence once structural and health‐related factors were considered.

Consistent with prior research, the most prominent reasons for non‐attendance were academic workload pressure and the availability of recorded lectures. These findings indicate that students prioritised ‘catching up’ and reliance on recordings as a substitute for in‐person teaching, reflecting the influence of structural academic pressures on attendance decisions. While lecture‐capture is widely valued for flexibility and convenience [11], it has also been associated with changes in attendance behaviour that may not support academic outcomes. This aligns with evidence that lecture‐capture availability can reduce attendance by encouraging substitution of live sessions [10] and encouraging students to skip more classes and postpone catching up [20, 23].

A crucial contextual factor is the institutional divergence in recording policies. KCL and SGUL operated institution‐wide lecture‐capture systems, while KUL did not. This pattern is consistent with institutional differences in lecture‐capture provision, suggesting that access to recorded teaching is a significant structural determinant of attendance decisions within this cohort. While recorded teaching may function as a simple substitute for attendance, the present findings support earlier studies in suggesting a more nuanced interpretation [11, 20]. The prominence of both recorded teaching and ‘being behind with studies’ as attendance‐related barriers indicates that students may use lecture capture strategically to manage academic workload, assessment pressures, commuting demands, and competing commitments. Consequently, reliance on recorded lectures should not necessarily be interpreted as disengagement from learning but may instead represent an alternative form of engagement through self‐paced study. The present study did not examine academic performance or patterns of lecture‐capture usage directly and therefore cannot determine whether the use of recorded teaching primarily reflects attendance substitution, workload management, or a combination of both.

The influence of journey length on barriers demonstrates that commuter‐related logistical constraints function as macro‐level structural determinants shaping attendance decisions [5, 6]. Students with medium‐length journeys (31–90 min) reported the highest academic strain, consistent with a ‘squeezed middle’ pattern. This finding suggests that commuter experience is not necessarily linear, with students experiencing moderate travel times potentially occupying a particularly challenging position. Such journeys may be sufficiently long to disrupt study routines and preparation, yet not long enough to prompt the adaptive behaviours or scheduling strategies often adopted by students facing more substantial commuting demands. While students with longer commutes (90+ minutes) may be more likely to rely on remote learning approaches, those with medium‐length journeys may be less able to balance commuting demands with pre‐session preparation. High‐ranking barriers such as ‘feeling behind’ are therefore consistent with structural constraints rather than individual factors, reflecting curriculum designs that may not fully account for the time demands associated with urban commuting.

Importantly, demographic characteristics (ethnicity, gender, age, year of study, Home/Overseas status) showed minimal or no independent influence on attendance barriers once structural factors such as journey length were accounted for. Ethnicity and fee status functioned largely as structural proxies for journey length; once travel time was controlled for, ethnic differences in attendance barriers were not observed. This aligns with research demonstrating that attendance patterns are shaped more strongly by institutional context and structural pressures than by demographic identity [24]. These findings contribute to wider discussions surrounding awarding and participation gaps by suggesting that apparent demographic differences may, in part, reflect exposure to unequal structural conditions rather than inherent differences between student groups [3, 4].

Health‐related effects were selectively but consistently linked to a limited number of barriers. Social anxiety, crowding, and fatigue were associated with increased reporting of social and interaction‐related attendance barriers, particularly reduced confidence in group work and peer‐related uncertainty. Prior research indicates that anxiety is a significant factor in higher‐education learning [25] and may be heightened by active learning demands [26], supporting the patterns observed here.

In contrast to attendance barriers, seating behaviour was primarily associated with health factors rather than academic or structural variables. Students reporting social anxiety or crowding sensitivity consistently selected peripheral, low‐density, or exit‐adjacent seating. This is consistent with previous evidence that students with anxiety preferentially select spatial positions that minimise sensory exposure, social evaluation, and visual intensity [18, 19, 27]. Such behaviours are likely individual‐level strategies to maintain perceived control and reduce social density within the learning environment [15, 28].

Demographic associations with seating behaviour were limited, with modest effects observed for age and gender and no consistent associations for ethnicity. Previous findings have suggested links between seating location and outcomes such as academic performance and motivation [14, 16, 29]; however, these outcomes were not examined in the present study, which instead identified stronger associations with psychological and sensory factors. This expands upon the seat‐mapping paradigms of Smith et al. [18], confirming that student spatial behaviour is deeply governed by micro‐level affective and sensory‐regulation choices rather than macro‐level logistics.

No significant institutional differences were observed in seating behaviour, despite clear institutional effects on attendance barriers. While institutional factors (such as lecture‐capture provision) were associated with attendance patterns, seating choice appeared to reflect responses to the immediate social and sensory environment. These findings indicate that attendance decisions are more sensitive to institutional context, whereas seating behaviour is more strongly associated with social and sensory conditions within the learning environment.

Collectively, these findings provide support for a dual‐dimensional view of student engagement, in which attendance and in‐class spatial behaviour reflect distinct but complementary aspects of interaction with the learning environment.

Across both strands of analysis, two consistent patterns were observed:

- Attendance barriers (macro‐level) were most strongly associated with academic workload, structural constraints (particularly commuting), and institutional infrastructure such as lecture capture provision [10]
- Seating behaviour (micro‐level) was most consistently associated with sensory and emotional factors, particularly social anxiety and sensitivity to crowding [18, 19]

This distinction suggests that students engage with different aspects of the learning environment through distinct behavioural responses. Attendance decisions appear to reflect responses to external academic and structural conditions, whereas seating behaviour reflects how students manage the immediate social and sensory environment of the classroom.

Health‐related factors were the only variables showing associations in both domains, indicating that some students may adopt both behavioural (e.g. selective attendance) and spatial (e.g. peripheral seating) strategies to manage their learning experience. This highlights the importance of considering both structural and sensory dimensions when interpreting student behaviour. The qualitative findings further strengthen these interpretations by demonstrating that students consistently describe attendance decisions in relation to workload management, commuting demands, academic pacing, and the perceived value of different teaching formats. The recurrence of these themes across focus groups drawn from multiple institutions supports the broader transferability of the proposed dual‐dimensional framework beyond individual campus contexts. A conceptual summary of the proposed dual‐dimensional framework is presented in Fig. 3.

**Fig. 3:**
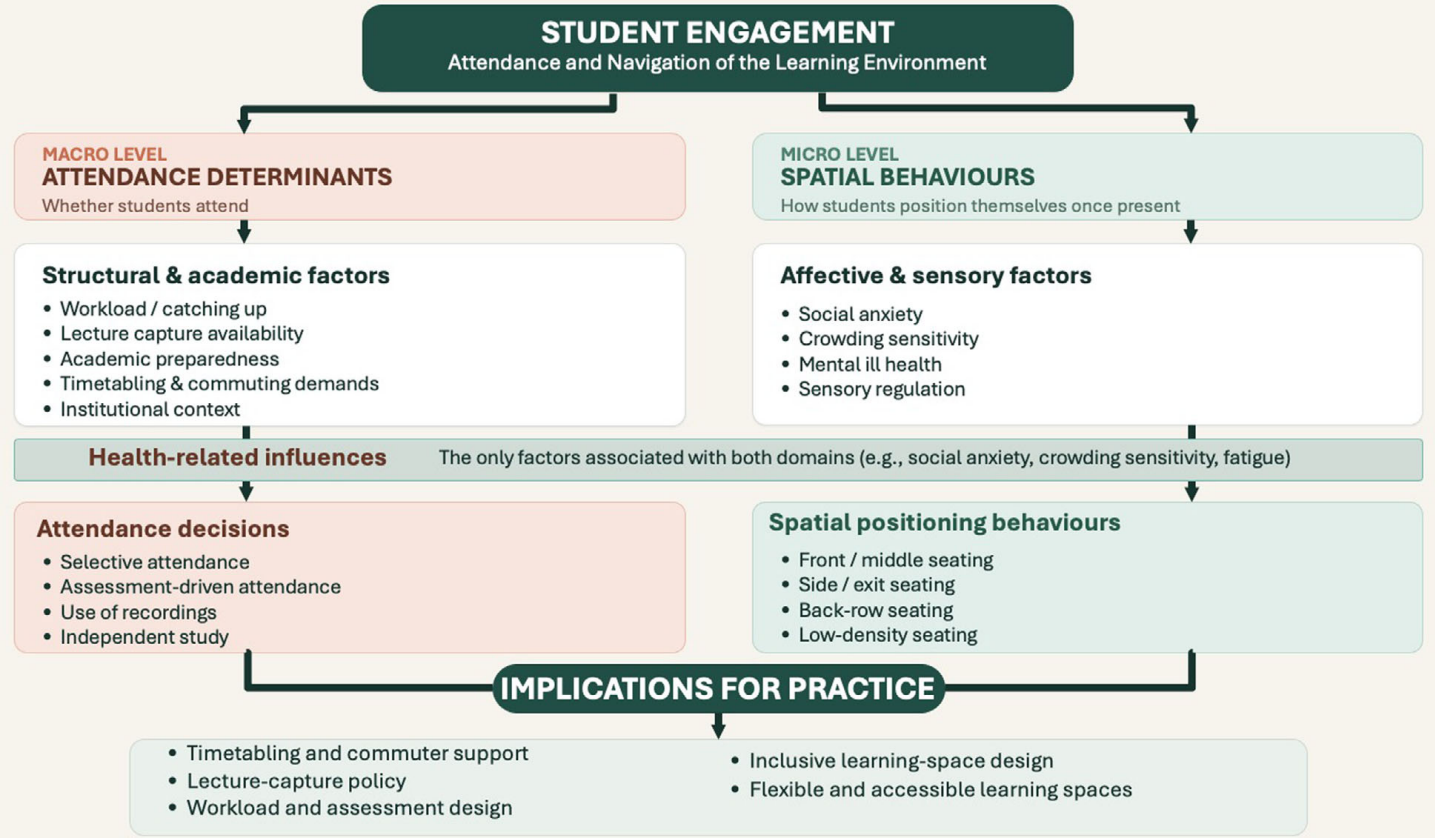
Dual‐dimensional model of undergraduate bioscience engagement.

## Implications

The findings of this study have several implications for bioscience teaching, particularly in large‐cohort and commuter‐dominated contexts.

First, the strong influence of academic workload and lecture‐capture availability on attendance highlights the importance of clearly communicating the role of recorded teaching. These findings suggest that institutional communication around lecture‐capture provision should emphasise its role as a supplementary resource rather than a substitute for in‐person engagement, given its observed association with attendance decision‐making. This may include aligning recorded material with preparatory or revision activities rather than positioning it as an equivalent alternative to live teaching. At the same time, recorded teaching may also function as a strategic mechanism through which students manage academic workload, assessment pressures, commuting demands, and competing commitments. Institutions should therefore consider how lecture‐capture provision supports flexible engagement while maintaining the distinctive benefits of live teaching. This consideration is particularly important given increasing recognition that student engagement cannot be understood solely through physical attendance [1]. For some students, including those managing health conditions, neurodiverse learning needs, caring responsibilities, financial pressures, or substantial commuting demands, recorded teaching may facilitate alternative forms of engagement and participation. Institutions should therefore seek to balance the benefits of in‐person learning with the inclusive opportunities afforded by flexible and digitally supported approaches.

Second, the association between journey length and academic readiness barriers emphasises the importance of timetabling and curriculum design that account for commuting constraints. In particular, the elevated strain observed among medium‐distance commuters suggests value in more consolidated timetables, reducing isolated or short‐duration sessions that require disproportionate travel effort relative to teaching time. Such approaches may help students in managing preparation and engagement with core content. More broadly, these findings support growing recognition that commuter experience should be considered within institutional approaches to student engagement, retention and success [7].

Third, the strong relationship between seating behaviour and social anxiety or crowding sensitivity indicates a role for learning‐space design in supporting inclusive engagement. Practical considerations include the availability of low‐density seating areas, flexible seating arrangements, and accessible exit‐adjacent locations, allowing students to manage comfort and participation within the lecture environment without disengaging entirely [28, 29]. These findings suggest that inclusive learning environments should not only focus on curriculum and pedagogy, but also consider how physical learning spaces accommodate diverse sensory and social needs. More broadly, these findings align with growing recognition that physical learning environments contribute not only to academic participation but also to student wellbeing, belonging, and psychological comfort [15].

Finally, the limited independent influence of demographic characteristics across both attendance and seating domains suggests that structural and environmental factors play a more consistent role in shaping engagement patterns than demographic categorisation alone. Interventions may therefore be most effective when focused on teaching practices, timetabling, digital provision, and learning environments that support a broad range of student needs, rather than targeting specific demographic groups.

Collectively, these findings have relevance beyond bioscience education and contribute to wider discussions within UK higher education concerning attendance, commuter support, inclusive practice, digitally supported learning, and how student engagement is conceptualised and measured. The proposed dual‐dimensional framework suggests that attendance decisions and in‐class participation behaviours may be driven by distinct structural and affective mechanisms. As institutions continue to expand blended and flexible models of delivery, recognising these different dimensions of engagement may support more effective policy development, curriculum design and student‐support strategies across a range of disciplinary and institutional settings.

## Limitations

Attendance barriers were measured using ranked self‐report items, capturing relative priority rather than absolute frequency or magnitude. As a result, lower‐ranked barriers should be interpreted as less influential in decision‐making at the time of response rather than absent. Furthermore, rankings do not reflect variation in the absolute strength of barriers between students; for example, one student's lower‐ranked barrier may have a greater overall impact on attendance than another's highest‐ranked barrier, potentially leading to misrepresentation of the overall significance of specific barriers. In addition, the cross‐sectional design limits causal interpretation; for example, feeling ‘behind’ may act as both a cause and a consequence of non‐attendance. Longitudinal approaches may help clarify the temporal relationships between these factors. Furthermore, the study did not examine relationships between attendance barriers, seating behaviour, and academic attainment. Consequently, while factors associated with engagement could be identified, their direct relationship with educational outcomes could not be determined, particularly within the anonymous design of the study. Future research linking engagement behaviours with academic performance may help clarify these relationships.

Seating behaviours were self‐reported as habitual behaviour rather than directly observed [18], which may introduce recall or reporting bias. While seating behaviour provided insight into how students interact with the physical learning environment, the study did not directly assess cognitive engagement, classroom participation, or academic performance. Consequently, seating location should be interpreted as a behavioural indicator of spatial interaction with the learning environment rather than a direct measure of student engagement. In addition, health‐related indicators were coded as binary variables and may mask variation in severity or intensity that could influence engagement behaviour.

While demographic characteristics showed limited independent associations once structural factors were considered, this should not be interpreted as indicating demographic irrelevance. Rather, it suggests that demographic differences may operate indirectly through structural factors, particularly commuting patterns, which were a primary focus of this study.

Finally, the sample was predominantly composed of female students aged 18–21 reflecting typical enrolment patterns within UK undergraduate bioscience programmes. While this may limit generalisability to more diverse cohorts, the sample remains representative of the disciplinary context examined.

## Conclusion

This study identifies and provides validation for a dual‐dimensional pattern of student engagement in bioscience education. Through the integration of quantitative findings and qualitative perspectives, we found that attendance decisions were associated with structural and academic factors, whereas in‐class spatial behaviour was more closely linked to sensory and emotional influences. Considering both dimensions highlights how students respond differently to the demands of the learning environment. These findings support a model in which student engagement is governed by distinct structural and affective mechanisms, with implications for curriculum design, lecture‐capture policy, and inclusive learning environments in large‐cohort bioscience education.

## Conflict of interest

The authors declare no conflict of interest.

## Author contributions

NP and AS conceived the study. NP, RJH, LJA and AS designed the study. NP, RJH, LJA, DB acquired, analysed, and interpreted the data. NP drafted the manuscript, and all authors contributed to editing and approved the final version. NP, RJH, and AS secured funding for the project.

## Supporting information


**File S1.** Survey instrument.

## Data Availability

All anonymised data analysed in this study are available from the corresponding author (NP) upon reasonable request.

## References

[1] Büchele S (2021) Evaluating the link between attendance and performance in higher education: the role of classroom engagement dimensions. Assess Eval High Educ 46, 132–150. doi: 10.1080/02602938.2020.1754330

[2] Lowe T , Naughton C , Struetzel T , Adams R , Walker J , Kynaston P and Mackey C (2025) Why are students not attending in‐person classes post‐COVID‐19? An explorative study in student engagement. Act Learn High Educ 0, doi: 10.1177/14697874251366213

[3] Gutman LM and Younas F (2025) Understanding the awarding gap through the lived experiences of minority ethnic students: an intersectional approach. Br Educ Res J 51, 990–1008. doi: 10.1002/berj.4108

[4] Li P‐H (2024) Student experience in HEIs: What does the evidence say about the ethnicity awarding gap? *The Diversity of Student Experience Project. The Centre for Teaching and Learning*. University of Oxford https://web.archive.org/web/20260421033819/https://www.ctl.ox.ac.uk/sitefiles/dse‐lit‐ethnicity‐april‐24.pdf

[5] Page N , Forster‐Wilkins G and Bonetzky M (2021) The impact of student timetables and commuting on student satisfaction. New Dir Teach Phys Sci 16, 2051–3615. doi: 10.29311/ndtps.v0i16.3793

[6] Hill J , Baird A , Wesley K , Tchibonkola J and Stewart‐Psaltis A (2024) Commuting students before and during COVID‐19: academic identity across home and campus spaces. Widening Particip. Lifelong Learn 26, 90–112. doi: 10.5456/WPLL.26.1.90

[7] Turner R , Rao N , Pritchard C , Webb O , Pinn E , Baharloo A and Ball H (2026) Supporting commuter students in UK higher education: opportunities to enhance policy and practice. Perspect Policy Pract High Educ 1–11, 1–11. doi: 10.1080/13603108.2026.2650337

[8] Alvarez‐Hevia D , Lord J and Naylor S (2021) Why don't they attend? Factors that influence the attendance of HE students of education. J Further High Educ 45, 1061–1075. doi: 10.1080/0309877X.2020.1851664

[9] Blair A and Clancy C (2024) Reviewing the complexities of student non‐attendance and the implications for block teaching. Stud Empowering Educ 5, 3. doi: 10.3943/gp.61

[10] Edwards MR and Clinton ME (2019) A study exploring the impact of lecture capture availability and lecture capture usage on student attendance and attainment. High Educ 77, 403–421. doi: 10.1007/s10734-018-0275-9

[11] Dommett EJ , Gardner B and van Tilburg W (2020) Staff and students perception of lecture capture. Internet High Educ 46, 100732. doi: 10.1016/j.iheduc.2020.100732

[12] Hillsey K (2026) Does recording lectures help? A within‐course availability comparison linking lecture capture and attendance to exam performance. Res Learn Technol 34, 3814. doi: 10.25304/rlt.v34.3814

[13] Page N , Hunt R , Albee L , Berwick D and Snape A (2026) Is it worth the journey? A multi‐institutional study of commuting, temporal agency, and on‐campus attendance in post‐pandemic London. SEHEJ (submitted)

[14] Shernoff DJ , Sannella AJ , Schorr RY , Sanchez‐Wall L , Ruzek EA , Sinha S and Bressler DM (2017) Separate worlds: the influence of seating location on student engagement, classroom experience, and performance in the large university lecture hall. J Environ Psychol 49, 55–64. doi: 10.1016/j.jenvp.2016.12.002

[15] Makaremi N , Yildirim S , Morgan GT , Touchie MF , Jakubiec JA and Robinson JB (2024) Impact of classroom environment on student wellbeing in higher education: review and future directions. Build Environ 265, 111958. doi: 10.1016/j.buildenv.2024.111958

[16] Meeks MD , Knotts TL , James KD , Williams F , Vassar JA and Wren OA (2013) The impact of seating location and seating type on student performance. Educ Sci (Basel) 3, 375–386. doi: 10.3390/educsci3040375

[17] Yu S , Zhang X , Tao R , Huang W , Chen J and Xiao X (2025) Effect of seating location on undergraduates' performance based on classroom size and quadrant surveying: a Chinese empirical analysis. SAGE Open 15, doi: 10.1177/21582440241310667

[18] Smith DP , Hoare A and Lacey MM (2018) Who goes where? The importance of peer groups on attainment and the student use of the lecture theatre teaching space. FEBS Open Bio 8, 1368–1378. doi: 10.1002/2211-5463.12494 PMC6120247 30186739

[19] Schussler E , Reynolds B , England B and Brigati J (2021) Student active learning anxieties differ by preferred seating location in introductory biology classes. J Coll Sci Teach 51, 67–77. doi: 10.1080/0047231X.2021.12290551

[20] Voelkel S , Bates A , Gleave T , Larsen C , Stollar EJ , Wattret G and Mello LV (2023) Lecture capture affects student learning behaviour. FEBS Open Bio 13, 217–232. doi: 10.1002/2211-5463.13548 PMC9900091 36610003

[21] Creswell JW and Plano Clark VL (2018) Designing and Conducting Mixed Methods Research. 3rd edn. SAGE Publications, California.

[22] Braun V and Clarke V (2012) Thematic analysis. In APA Handbook of Research Methods in Psychology, Vol. 2. Research Designs: Quantitative, Qualitative, Neuropsychological and Biological ( Cooper H , Camic PM , Long DL , Panter AT , Rindskopf D and Sher KJ , eds), pp. 57–71. American Psychological Association, Washington. doi: 10.1037/13620-004

[23] Horlin C , Hronska B and Nordmann E (2024) I can be a “normal” student: the role of lecture capture in supporting disabled and neurodivergent students' participation in higher education. High Educ 88, 2075–2092. doi: 10.1007/s10734-024-01201-5

[24] Loughlin C (2026) Building higher education: the tension between espoused educational values and physical infrastructure. High Educ 91, 369–386. doi: 10.1007/s10734-025-01421-3

[25] England B , Brigati J and Schussler E (2017) Student anxiety in introductory biology classrooms: perceptions about active learning and persistence in the major. PLoS One 12, doi: 10.1371/journal.pone.0182506 PMC5542638 28771564

[26] Cooper K , Downing V and Brownell S (2018) The influence of active learning practices on student anxiety in large‐enrollment college science classrooms. Int J STEM Educ 5, 23. doi: 10.1186/s40594-018-0123-6 PMC6310416 30631713

[27] Rahman M , Rambely A , Halim L and Mansor A (2025) The position of seats and its impact on anxiety among students. Int J Acad Res Prog Educ Dev 14, 457–465. doi: 10.6007/IJARPED/v14-i2/24361

[28] Totusek PF and Staton‐Spicer AQ (1982) Classroom seating preference as a function of student personality. J Exp Educ 50, 159–163. doi: 10.1080/00220973.1982.11011818

[29] Putman R , Pennington C , Munsey B , Hurless N , Hill JW and Labansat HA (2024) Does this chair make me look smart? An examination of flexible seating in the higher education classroom. Learn Environ Res 27, 893–911. doi: 10.1007/s10984-024-09509-w

