# Supplementary material for: Determinants of undergraduate bioscience engagement: Validating attendance barriers and classroom spatial behaviour across institutions
Source: FEBS Open Bio. 2026 Oct 3:10.1002/2211-5463.70352. Online ahead of print. doi: 10.1002/2211-5463.70352 (PMC13633484; doi:10.1002/2211-5463.70352)
Supplement: Supplementary file 1 [file FEB4-9999-0-s001.pdf]

Supplementary Information

Survey

Understanding and defining student engagement in the post-COVID-19 learning environment: a collaborative study.

The items presented below represent a subset of a broader survey examining student engagement in the post-COVID-19 learning environment. To maintain a focused analysis of attendance factors and classroom seating behaviours, items relating to structural and logistical barriers (e.g. travel, cost, and transport) have been excluded from this appendix. The full survey instrument is available from the authors upon request.

1. **How long does your typical journey to campus take you?**

0-15 mins	16-30 mins	31 mins-1 hour	1-1.5 hours
1.5-2 hours	2-2.5 hours	2.5-3 hours	More than 3 hours

2. **Regarding your learning experience, if you did not attend some on-campus teaching sessions, what were the reasons for this? Select up to three that were your main reasons.**

I have had that lecturer before and did not think they were very good or liked them	My friends were good at persuading me to do something else
None of my friends would be attending the session	Sessions were going to be recorded, and therefore I could catch up later
I did not know anyone who would be attending the session	I had not looked through and prepared for the session in advance
I felt academically behind/unprepared for the session	I find the pace of sessions too fast or too slow
These sessions are insufficiently interactive	I was not confident taking part in the group work
I do not enjoy that particular subject	I was not enjoying/interested in my course/did not feel motivated by my course
I felt that I already understood the subject well	I was behind with my studies and felt I needed to prioritise catching up
I prefer to learn through independent study	None of the above (not applicable)
Other reason, please specify (open text box)	

3. **When you attend an on-campus session where do you sit in the classroom / lecture theatre?**

Always/usually at the front	Always/usually in the middle
Always/usually at the back	Always/usually at the side or exit

Section with the fewest people	Wherever my friends sit – it varies
There is no typical location in which I sit	

4. Health reasons, why I could not attend some of my on-campus sessions. Tick all that apply.

I was experiencing physical ill health	I was experiencing mental ill health
I was too tired	I was still worried about catching COVID-19
I have a disability that makes it difficult to come to campus and/or access my class	Medical/dental appointments
I am anxious with other people in my lecture/don't like crowded environments	Other reason, please specify (open text box)
None of the above (not applicable)	

5. **Which gender do you identify as**?

Female	Male	Non-binary	Prefer not to say

6. What is your age?

Under 18	18-21	22-25	26-35	Over 36

7. What is your ethnicity?

Asian (including Chinese)	Black	Other (including mixed race)	White	Prefer not to say

8. Which university tuition fees do you pay?

Home	Overseas
